# Plasma Cell-Free Human Papillomavirus DNA and Oral Gargle HPV DNA in Patients with HPV-Related Oropharyngeal Cancer Treated with Radiotherapy

**DOI:** 10.1158/2767-9764.CRC-25-0180

**Published:** 2025-07-22

**Authors:** Michelle Echevarria, Robin Park, Jimmy J. Caudell, Youngchul Kim, George Q. Yang, Kedar Kirtane, Ritu Chaudhary, Sunil Kumar, Antonio L. Amelio, Anna R. Giuliano, Christine H. Chung

**Affiliations:** 1 Department of Radiation Oncology, Moffitt Cancer Center, Tampa, Florida.; 2 Department of Head and Neck-Endocrine Oncology, Moffitt Cancer Center, Tampa, Florida.; 3 Department of Biostatistics and Bioinformatics, Moffitt Cancer Center, Tampa, Florida.; 4 Naveris, Inc., Waltham, Massachusetts.; 5 Department of Tumor Microenvironment and Metastasis, Moffitt Cancer Center, Tampa, Florida.; 6 Department of Cancer Epidemiology, Moffitt Cancer Center, Tampa, Florida.

## Abstract

Dynamic biomarkers that guide de-escalation strategies in human papillomavirus (HPV)-related oropharyngeal squamous cell carcinoma (OPSCC) remain an unmet need. In this study, we evaluated the kinetics of plasma cell-free HPV (cfHPV) DNA and oral gargle HPV DNA during radiotherapy in patients with low-risk HPV-related OPSCC. Data were obtained from a trial evaluating an adaptive model optimizing radiation fractionation in patients with low-risk (T0-2N0-1M0) HPV-related OPSCC undergoing radiotherapy. The primary objective was to determine whether week 4 plasma cfHPV DNA or oral gargle HPV DNA clearance is associated with reduction of target tumor volume (TTV) at week 4. A total of 325 plasma and 334 oral gargle samples from 50 patients with available baseline samples were analyzed. Higher baseline plasma cfHPV DNA was associated with higher nodal staging (*P* = 0.002), whereas oral gargle HPV DNA was detected more frequently in the tonsil or soft palate than occult or base of tongue primary tumors (*P* = 0.039). Week 4 plasma but not oral gargle HPV DNA clearance was associated with higher reduction of TTV at week 4 (*P* = 0.0063). Whereas week 4 plasma and oral gargle HPV DNA clearance was not associated with progression-free survival, a lower baseline plasma cfHPV DNA was associated with superior progression-free survival (*P* = 0.027). Week 4 plasma cfHPV DNA clearance aligns with reduction in TTV, and future studies are warranted to determine the role of early plasma cfHPV DNA clearance in biomarker-adapted de-escalation strategies.

**Significance:**

Our findings may inform appropriate patient selection for low-risk HPV-related OPSCC based on cfHPV DNA in future deintensification studies, aimed at preventing or minimizing treatment-related toxicities in patients who may have lower risk of recurrence.

## Introduction

The incidence of human papillomavirus (HPV)-related oropharyngeal squamous cell carcinoma (OPSCC) has increased substantially over the past three decades, accounting for 71% of all newly diagnosed OPSCC cases in the United States (1–4). The standard treatment for patients with curable HPV-related OPSCC includes definitive radiotherapy (RT) with or without concurrent chemotherapy or surgical resection followed by adjuvant therapy (5). As HPV-related OPSCC is generally associated with higher cure rates and longer overall survival (OS) than HPV-unrelated head and neck squamous cell carcinoma (HNSCC), there has been considerable interest in the deintensification of treatment aimed at reducing the burden of long-term treatment-related toxicities while maintaining the efficacy of oncologic outcomes. However, randomized trials evaluating deintensification strategies, such as substituting cisplatin with cetuximab for concurrent chemoradiation or reducing the dose of radiation delivered based solely on HPV status, have shown inferior clinical outcomes, underscoring the need for more refined, biomarker-driven strategies (6–8). Thus, the development of novel biomarkers to improve patient selection for deintensification strategies in HPV-related OPSCC remains an area of unmet need.

Tumor-tissue modified (TTMV) HPV DNA (NavDx) is a prognostic biomarker that uses digital droplet PCR (ddPCR) to detect plasma cell-free HPV (cfHPV) DNA in HPV-related OPSCC. In the clinical setting, plasma cfHPV DNA may be used as a prognostic biomarker to guide surveillance after curative-intent treatment, as well as to monitor disease activity in conjunction with radiographic imaging in recurrent and/or metastatic HNSCC (9). In addition, plasma cfHPV DNA has also been explored as a potential dynamic on-treatment biomarker of therapeutic response in curative-intent treatment settings through retrospective and prospective research studies (10–13). For example, clearance of plasma cfHPV DNA after definitive chemoradiation and induction chemotherapy has been associated with superior progression-free survival (PFS; refs. 12, 13). However, the kinetics of plasma cfHPV DNA measured during treatment in patients with HPV-related OPSCC undergoing definitive RT has not been well characterized, and its use as an on-treatment biomarker remains unclear.

HPV DNA may also be detected in oral gargle samples. Prior studies have shown that oral gargle may be superior to other noninvasive sampling methods, such as brushings of the oropharynx, in detecting HPV infections in patients with nonmalignant oropharyngeal lesions (14). In addition, oral gargle samples can be used to detect clinically relevant high-risk HPV genotypes in patients with OPSCC, among whom the persistence of oral gargle HPV DNA after the completion of definitive treatment has been associated with inferior 2-year OS and recurrence-free survival (15–17). Therefore, given its noninvasiveness, cost-effectiveness, and ease of measurement, oral gargle HPV DNA testing represents a potential dynamic biomarker for treatment modification as a part of deintensification treatment strategies. Thus, in addition to plasma, oral gargle HPV DNA testing warrants further evaluation as a potential prognostic and predictive biomarker in patients undergoing curative treatment for HPV-related OPSCC.

Previously, we reported findings from a phase II clinical trial evaluating the feasibility of optimizing curative-intent RT using the prognostic scoring index, a mathematical model of pretreatment growth and radiation response dynamics, in patients with low-risk early-stage HPV-related OPSCC (18). In this study, patients with HPV-related OPSCC received either conventional fractionation (2 Gy daily to a total dose of 60–70 Gy) or hyperfractionated radiation (1.2 Gy twice daily to a total dose of 60–69.6 Gy) without chemotherapy depending on their pretreatment prognostic scoring index scores. The rate of early target tumor volume (TTV) reduction of ≥32% at 4 weeks was 61.2%. The complete response rate based on end-of-treatment imaging at 2 to 3 months after treatment was 98.1%. The all-cause mortality was 3.64% (2/55). The rate of serious adverse events was 1.82% (1/55). As part of the exploratory analysis, plasma and oral gargle biospecimens were collected at predefined intervals for cfHPV DNA analysis. In this study, we report the analysis of plasma cfHPV DNA and oral gargle HPV DNA in participants enrolled in a clinical trial, in which we aimed to assess the association of HPV DNA clearance in the plasma and oral gargle with early on-treatment and end-of-treatment responses.

## Materials and Methods

### Study design and patients

Patient data used in this study were collected from a prospective single-center clinical trial (NCT03656133) that enrolled patients from August 2018 to July 2022. The details of the clinical trial, including the interim results, are presented in the European Society of Radiotherapy and Oncology 2023 (18). Briefly, the primary objective of this parallel cohort clinical trial was to evaluate the feasibility of using a pretreatment mathematical model of cell growth dynamics to inform radiation fractionation, in which patients were assigned to one of two parallel cohorts: conventional fractionation to a total dose of 60 to 70 Gy in 2 Gy per fraction or hyperfractionation to a total dose of 60 to 69.6 Gy in 1.2 Gy per fraction delivered twice daily. The key inclusion criteria of the study were patients with T0-2, N0-1, and M0-p16–positive OPSCC, in which T is tumor stage, N is node stage, and M is metastasis stage, based on the American Joint Committee on Cancer eighth edition staging. Notably, in addition to patients with N1 disease with a single node ≤3 cm, patients with 2 to 5 involved ipsilateral lymph nodes or a single involved lymph node measuring >3 cm were eligible for enrollment if the number of pack-years smoked was ≤10. The rationale for this inclusion criterion was based on the exploratory analysis of the RTOG 1016 trial, which showed that the number of pack-years smoked >10 was associated with greater benefit from the addition of chemotherapy to radiation, and an unpublished retrospective analysis conducted at Moffitt Cancer Center, demonstrating that patients with 2 to 5 involved nodes had comparable 3-year locoregional disease-free survival to patients treated with chemoradiation if the number of pack-years smoked was ≤10^7^. Key exclusion criteria included patients with more than five involved lymph nodes, distant metastatic disease, prior RT to the region of the cancer that would result in overlap of RT fields, or gross total excision of both primary and nodal disease.

This study was conducted in accordance with the Declaration of Helsinki ethical principles, Good Clinical Practices, principles of informed consent, and the requirements for public registration of clinical trials. Written informed consent forms describing the study procedures and risks were given to the participants, and signed informed consent forms were obtained prior to starting intervention or conducting any study procedures. The study protocol was approved by the Institutional Review Board of Moffitt Cancer Center. Supplementary Table S1 shows the representativeness of study participants.

### Clinical outcomes

The primary objective of this study was to evaluate the association between week 4 plasma cfHPV DNA and oral gargle HPV DNA clearance and early on-treatment (week 4) TTV reduction assessed via local investigator assessment. The tumor volume was inclusive of both primary tumor and involved lymph nodes. Of note, the primary endpoint of the clinical trial was the proportion of patients with TTV reduction of ≥32% at week 4, the rationale of which is based on prior data showing that achievement of TTV of ≥32% at week 4 was associated with superior 1-year locoregional control and disease-free survival rates in patients with HPV-related OPSCC undergoing definitive radiation with or without chemotherapy in a retrospective analysis (19). Therefore, we compared the % reduction of TTV at week 4 between patients who did versus did not achieve cfHPV DNA clearance at week 4. Week 4 CT and/or MRI were loaded into the treatment planning system or other software for volumetric evaluation of tumor size and were compared against CT simulation or week 1 CT and/or MRI, whichever was larger. The percentage of responses was then calculated.

### Collection of plasma and oral gargle biospecimens

cfHPV DNA analysis was conducted as a *post hoc* analysis using biospecimens collected as a part of the trial protocol (Supplementary Fig. S1). Blood samples were collected in 10-mL EDTA collection tubes. Plasma was processed, approximately 3 mL of plasma was extracted, and sample quality was assessed by performing a ddPCR assay targeting a region of the *ESR1* gene. Plasma samples were collected at baseline, at week 4 during RT, at the end of treatment, and after treatment (2–3, 6, and 12 months). Oral gargle samples were obtained by performing a 30-second rinse and gargle with 15 mL of mouthwash and collected using 50-mL conical tubes. The samples were processed as previously described (20). Oral HPV DNA was extracted using an automated BioRobot MDx kit (Qiagen; RRID: SCR_020412). Oral gargle samples were collected at baseline, weekly during RT, at the end of treatment, and after treatment (2–3, 6, and 12 months).

### cfHPV DNA analysis in plasma and oral gargle biospecimens

cfHPV DNA in the plasma samples was assessed using the NavDx assay (Naveris, Inc.; ref. 21). The NavDx assay quantifies TTMV-HPV DNA using ddPCR coupled with algorithmic analysis of tumor-specific HPV DNA fragmentation patterns to detect tumor-derived HPV DNA and reports results from five high-risk HPV types (16, 18, 31, 33, and 35) as a TTMV-HPV DNA score. The TTMV-HPV DNA score, which is mathematically adjusted to account for plasma volume, indicates the likelihood that a patient has HPV-related cancer and can distinguish between tumor-derived fragmented HPV DNA and native HPV genomic DNA, regardless of tumor HPV integration status. For oral gargle specimens, HPV genotype was determined using the RHA Kit HPV SPF10-LiPA25 (DDL Diagnostic Laboratory), which is an *in vitro* reverse hybridization assay for the qualitative identification of HPV DNA. LiPA25 targets a 65-base pair fragment of the L1 region of the HPV genome. This assay requires a three-step process as previously described (22). Oncogenic HPV types included HPV 16, 18, 31, 33, 35, 39, 45, 51, 52, 56, 58, 59, and 68. Positive high-risk results were considered as types 16, 18, 31, 33, and 35. This assay has proven to detect up to 20% more HPV types than other marketed assays, making it the most sensitive assay for samples with low copy number and/or mixed-type HPV samples like those obtained in oral gargles (23, 24).

### Statistical analysis

The correlation between cfHPV DNA clearance in either plasma or oral gargle biospecimens at specific time points during on-treatment and imaging-assessed treatment response at on-treatment and after treatment was assessed using the *χ*^2^ test. Time-to-endpoint outcomes such as PFS were assessed using the Kaplan–Meier method, and between-group comparisons were assessed using the log-rank test. Correlations were assessed using Pearson’s correlation coefficient. Statistical significance was set at *P* < 0.05. Analyses were performed using R version 4.4.1 (RRID: SCR_001905).

### Data availability

Data generated in this study are available upon request from the corresponding author.

## Results

### Patient characteristics and clinical outcomes

**Figure 1:**
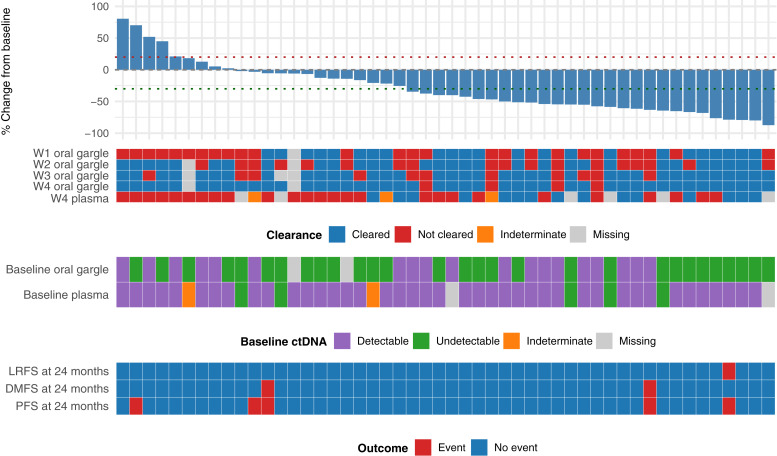
Waterfall plot showing the % change of week 4 TTV compared with baseline for all patients who met the inclusion criteria (*N* = 50). Each legend at the bottom of the waterfall plot show the status of HPV DNA clearance for oral gargle at weekly intervals for weeks 1 to 4 and for plasma at week 4; baseline HPV DNA status by plasma and oral gargle; and the locoregional disease-free survival (LRFS), distant metastatic disease-free survival (DMFS) recurrence, and PFS statuses at 24 months after treatment of individual patients.

The median follow-up period was 41 months [95% confidence interval (CI), 33–48] with a data cutoff date of May 20, 2024. Baseline patient characteristics are summarized in Table 1. Of the 55 patients enrolled in the trial, 50 patients met the inclusion criteria, of whom 46 had both plasma and oral gargle, 2 had only plasma, and 2 had only oral gargle for HPV DNA analyses (Fig. 1). At posttreatment imaging, one patient was found to have a vertebral metastasis; this was evident but not identified by the radiology report on pretreatment imaging. The complete response rate at end-of-treatment imaging was 96% (48/50). At the time of data cutoff, the median PFS and OS were not reached. The 2-year PFS rate was 89.8% (95% CI, 81.7–98.7), and the 2-year distant metastatic and locoregional disease-free survival rates were 95.6% (95% CI, 89.9–100) and 97.9% (95% CI, 93.8–100), respectively.

**Table 1:** Baseline patient characteristics

Patient characteristics	*N* (range)
Number of patients	50
Median age	64 (30–82)
Sex	​
Female	9
Male	41
ECOG status	​
0	41
1	9
Smoking status	​
Never smoker	26
Current/former smoker	24
Primary tumor location	​
Base of tongue	29
Tonsil	19
Soft palate	1
Unknown primary	1
T stage	​
T0	1
T1	28
T2	21
N stage	​
N0	5
N1	45
Number of tumor-involved lymph nodes	​
0	5
1	20
2	19
3	4
4	2
High-risk HPV genotypes (plasma)	​
Type 16	39
Type 33	2
Indeterminate/not available	7
High-risk HPV genotypes (oral gargle)	​
Type 16	19
Type 18^a^	1
Type 35^a^	1
Indeterminate/not available	29

a One patient had concomitant type 16 and type 18, and one patient had concomitant type 16 and type 35.

Abbreviations: ECOG, Eastern Cooperative Oncology Group; N, node; T, tumor.

### cfHPV DNA detection in plasma biospecimens

A total of 325 plasma samples were analyzed. We determined whether there was an association between plasma cfHPV DNA detectability and the selected baseline clinical characteristics. A total of 82% (41/48) of patients had cfHPV DNA detectable in their baseline plasma specimens, with the majority comprising HPV-16, followed by HPV-33 (Table 1). HPV RNA *in situ* hybridization was available in 4 of 7 patients who had undetectable or indeterminate baseline plasma cfHPV DNA, all four of whom were HPV-16 positive. Of the remaining patients with unknown HPV RNA *in situ* hybridization (3/7), one developed positivity for HPV-16 in the plasma at week 4, whereas another had an initially negative oral gargle cfHPV DNA and developed positivity for HPV-33 at week 1. The median TTMV-HPV DNA score among patients with detectable baseline plasma cfHPV DNA was 132 (range, 2–4,366). Absence of any smoking history (*P* = 0.019) was associated with detectable baseline plasma cfHPV DNA levels. Higher nodal staging was associated with detectable baseline plasma cfHPV DNA (Supplementary Fig. S2A). Additionally, higher median baseline plasma cfHPV DNA was found in patients with higher number of involved nodes (0–1 involved nodes, 33; ≥2 involved nodes, 4,008). The baseline T staging and the primary tumor location demonstrated no association with baseline plasma cfHPV DNA detectability (Supplemental Fig. S2A).

**Figure 2:**
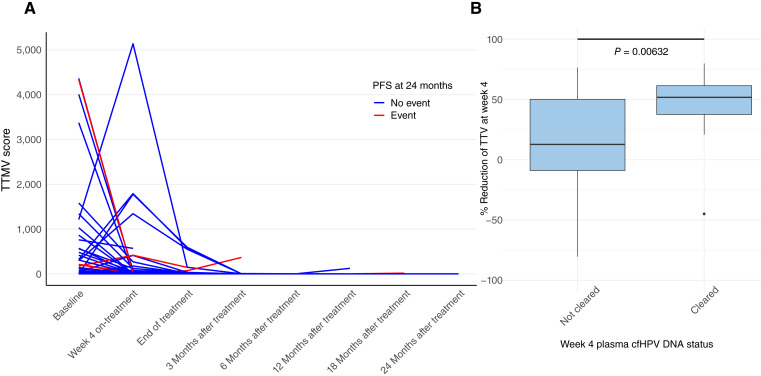
Dynamic changes of plasma cfHPV DNA and its association with reduction of TTV at week 4. **A,** Plasma cfHPV DNA kinetics before, during, and after definitive radiation treatment. Each colored line represents a patient. Colors of lines represent patients who did (red) and did not (blue) have confirmed disease progression at 24 months. **B,** A box plot showing the % reduction of TTV at week 4 for patients who did or did not achieve plasma cfHPV DNA clearance at week 4. Positive (i.e., >0) numbers represent tumor volume reduction, whereas negative (i.e., <0) numbers represent tumor volume increase. Statistical test represents the Mann–Whitney test between the two groups overlaid by the bolded line. The median and upper and lower quartiles are represented in the bar graphs.

**Figure 3:**
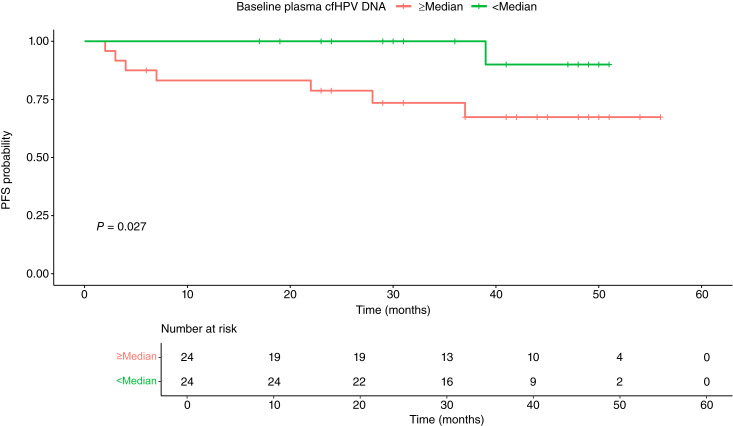
Association between baseline cfHPV DNA and PFS. Comparison of PFS for patients with low (<median) versus high (<median) baseline plasma cfHPV DNA. Log-rank test represents a comparison between the respective groups.

We then analyzed the association between plasma cfHPV DNA and selected treatment outcomes. Among patients with detectable baseline plasma cfHPV DNA, 39% (16/41) had clearance to undetectable levels by week 4 of RT (Fig. 1). Graphically, no differences were readily apparent in the kinetics of plasma cfHPV DNA between patients who were versus were not progression-free at 24 months from end of treatment (Fig. 2A). Subsequently, 66.7% (26/39) and 97.4% (37/38) of the patients who had any prior detectable plasma cfHPV DNA levels had clearance at the end of treatment and at 3 months after treatment, respectively. Additionally, 97.5% (39/40) and 96.7% (29/30) of the patients with available plasma samples for analysis had negative plasma cfHPV DNA at 6 and 12 months after treatment, respectively. A total of four patients had detectable plasma cfHPV DNA levels beyond end of treatment. One patient with positive cfHPV DNA at 3 months had distant metastasis detected at 3 months; the patient with positive cfHPV DNA at 6 months had locoregional recurrence at 7 months; and the patient with positive cfHPV DNA at 12 months had locoregional recurrence at 28 months. The remaining patient is progression-free at the time of data cutoff. No correlation was found between week 4 TTV reduction and week 4 plasma cfHPV DNA (*R*^2^ = 0.031, *P* = 0.239); however, week 4 plasma cfHPV DNA clearance was associated with higher % reduction of TTV at week 4 (*P* = 0.0063; Fig. 2B). Whereas plasma cfHPV DNA clearance at week 4 was not associated with a statistically significant difference in PFS, lower (<median) baseline plasma cfHPV DNA had a statistically significant association with superior PFS (log-rank, *P* = 0.027; Supplementary Fig. S3A; Fig. 3). We then analyzed the clinical outcomes of a patient subset who had a transient spike in plasma cfHPV DNA at week 4, which we defined as any increase compared with the baseline. In this subgroup of patients (*N* = 8), all patients had complete anatomic and metabolic responses, and 87.5% (7/8) were progression-free at 2 years.

### cfHPV DNA detection in oral gargles biospecimens

A total of 334 oral gargle samples were analyzed. We determined whether there was an association between oral gargle HPV DNA detection and selected baseline clinical characteristics. Baseline oral gargle HPV DNA was detected in 39% (19/48) of the patients, all of whom showed HPV type 16 (Table 1; Fig. 1). One patient had concurrent HPV-35, and another had concurrent HPV-18. Patients with tonsil or soft palate primary tumor sites were more likely to have detectable baseline oral high-risk HPV than those with base of tongue or occult primary tumor sites (*P* = 0.039; Supplementary Fig. S2B). No association was found among the baseline T staging, N staging, number of involved nodes, smoking status, and detectable baseline oral gargle HPV DNA (Supplementary Fig. S2B). Patients who had positive baseline HPV DNA levels in the oral gargle were more likely to have positive baseline HPV DNA in plasma samples (*P* = 0.021; Fig. 1).

Next, we analyzed the association between oral gargle HPV DNA and selected treatment outcomes. Notably, 41.3% (12/29) of the patients with negative baseline oral gargle HPV DNA were positive during weeks 1 to 3 of RT. Clearance of oral gargle HPV DNA was demonstrated at week 4 in 87% (27/31) of patients who had any prior detectable levels. Subsequently, 96.7% (30/31) and 92% (23/25) of patients with any prior detectable oral gargle HPV DNA had clearance at the end of treatment and at 3 months after treatment, respectively. In addition, 96.8% (30/31) and 95.8% (23/24) of patients with available plasma samples tested negative for oral gargle HPV DNA at 6 and 12 months after treatment, respectively. No association was found between oral gargle HPV DNA clearance and % reduction of TTV at week 4 (*P* = 0.391; Supplementary Fig. S4A). Based on a review of our data, we reasoned that HPV DNA seemed to clear earlier than in the plasma, and we analyzed the association between early TTV reduction at week 4 and oral gargle HPV DNA collected at earlier time points. However, we did not observe a significant association between higher early TTV reduction and oral gargle HPV DNA clearance at weeks 1 (*P* = 0.517), 2 (*P* = 0.185), and 3 (*P* = 0.702; Supplementary Fig. S4B–S4D).

PFS was not compared between patients who had clearance versus those who had no clearance of week 4 oral gargle HPV DNA because the proportional hazards assumption was violated (Supplementary Fig. S3B). We also analyzed the clinical outcomes of a patient subset with undetectable baseline oral gargle HPV DNA that subsequently turned positive after starting treatment. In this subgroup of patients (*N* = 12), all patients had complete anatomic and metabolic responses and 91.7% (11/12) were progression-free at 2 years.

### Concordance between plasma and oral gargle cfHPV DNA

We analyzed whether plasma cfHPV DNA and oral gargle HPV DNA samples showed concordance in clearance at week 4 of RT in patients with detectable levels of plasma cfHPV DNA and oral gargle HPV DNA at baseline (*N* = 18; Fig. 1). Among these patients, 38.9% (7/18) cleared HPV DNA by week 4 in both the plasma and oral gargle samples. However, 55.6% (10/18) of patients had discordant results between the plasma cfHPV DNA and oral gargle HPV DNA samples, with a significantly higher proportion of patients having cleared HPV DNA by week 4 in oral gargle but not in plasma compared with patients who cleared in plasma but not in oral gargle (Fisher exact test, *P* = 0.015). In addition, the baseline high-risk type detected was HPV-16 in the plasma and oral gargle in all patients, although 11.1% (2/18) of patients had codetection of an additional high-risk HPV type in the oral gargle. Of note, patients who had detectable HPV-33 in the plasma at baseline (*N* = 2) had undetectable HPV DNA in the oral gargle at baseline. Subsequently, we analyzed the association between patients who had a transient week 4 increase in plasma cfHPV DNA and those who had baseline undetectable oral gargle HPV DNA levels that later became detectable after treatment. Among the eight patients with transient week 4 plasma cfHPV DNA increase and 12 patients with baseline negative oral gargle HPV DNA that later became detectable, only two patients had both events, suggesting low concordance between these phenomena.

## Discussion

As part of exploratory analyses within a completed phase II trial, we evaluated the potential role of plasma cfHPV DNA and oral gargle HPV DNA as early dynamic response biomarkers in patients with low-risk HPV-related OPSCC undergoing definitive RT. Our data demonstrated that early (week 4) plasma cfHPV DNA clearance was associated with higher % reduction of TTV at week 4 of RT. Furthermore, our results show that oral gargle HPV DNA clearance was not associated with reduction of TTV at week 4 of RT and indicate that oral gargle has poor correlation with radiation treatment response. In addition, we report novel insights into the kinetics of plasma and oral gargle HPV DNA during and after definitive RT in patients with low-risk HPV-related HNSCC.

Prior studies have demonstrated that pretreatment plasma cfHPV DNA levels are more closely associated with the extent of nodal involvement rather than the primary tumor volume (11–13, 25, 26). Our results mirror these prior findings, showing that higher baseline plasma cfHPV DNA levels are associated with a higher number of involved lymph nodes and not a higher baseline T staging. HPV DNA oral gargle was tested using the highly sensitive SPF(10) assay, which has demonstrated superior sensitivity to the linear array and has been used in the HPV-16/18 AS04-adjuvanted vaccine trial (27, 28). For oral gargle samples, we observed a statistically nonsignificant trend toward an association with larger primary tumor volumes and detectable HPV DNA at baseline. Furthermore, oral gargle HPV DNA was more readily detected at baseline in patients with primary tumors of the tonsil or soft palate than in those with occult primary or base of tongue tumors. As the oral gargle obtains cells exfoliated into the oral cavity and throat, cells from the base of tongue may be less likely to be represented in the oral gargle compared with the tonsils and hence a lower rate of HPV DNA detection if the tumor is at the base of tongue.

The low sensitivity of baseline plasma cfHPV DNA in our study is consistent with previously reported studies of HPV-related HNSCC (29, 30). We observed that lower baseline plasma cfHPV DNA (<median) was associated with superior PFS in the participants of our study. Our findings are at odds with the results of Chera and colleagues (12), who concluded that lower baseline plasma cfHPV DNA levels are associated with worse prognosis as they are associated with higher likelihood of HPV DNA integration, which in turn has been associated with adverse clinical outcomes in HPV-related OPSCC (31–33). However, in their study, patients with T3 or T4 primaries had statistically significantly lower levels of plasma cfHPV DNA compared with those with T2 primaries, suggesting that the association between lower baseline plasma cfHPV DNA levels and inferior outcomes may have been driven by patients with more advanced disease. Supporting this notion, in Koneva and colleagues (32), HPV DNA integration was observed more frequently in patients with more advanced clinical stages in their in-house cohort comprised predominantly of HPV-related OPSCC (AJCC seventh edition, stage I–III, 0% vs. IV, 60%). In the context of these studies, our findings show that lower baseline plasma cfHPV DNA levels in early-stage (T0-2N0-1M0) OPSCC are associated with favorable prognostic outcomes, suggesting baseline plasma cfHPV DNA may guide patient selection to guide RT adaptation. Radiation dose deintensification for locoregionally advanced nasopharyngeal cancer for patients with lower (<4,000 copies/mL) Epstein–Barr virus titers has shown favorable survival outcomes and safety in a phase II trial (34). Such a study design in Epstein–Barr virus-related nasopharyngeal cancer may also be applicable in future studies of early-stage HPV-related OPSCC evaluating personalized treatment strategies using baseline cfHPV DNA levels in patients undergoing definitive RT. Indeed, ongoing studies are evaluating the role of HPV kinetics in treatment deintensification (NCT04900623, NCT03215719, and NCT05268614).

Early plasma cfHPV DNA clearance has been associated with superior clinical outcomes in patients with HPV-related OPSCC in various treatment settings, including neoadjuvant combined chemoimmunotherapy, chemoradiation, and induction chemotherapy (10, 12, 13, 26). Our study adds to the literature by reporting on the kinetics of plasma cfHPV DNA in patients undergoing definitive RT alone. We observed that plasma cfHPV DNA clearance at week 4 during RT was associated with higher % reduction of TTV at week 4, which is a surrogate marker of improved locoregional control (35). Whereas we found no association between early plasma cfHPV DNA clearance at week 4 of RT and superior PFS, our analysis was limited by a short-term follow-up with insufficient number of events due to the excellent prognoses of the trial participants who have low-risk HPV-related OPSCC. Furthermore, analysis of the association between plasma cfHPV DNA and oral gargle HPV DNA and end-of-treatment response was not feasible, as most patients achieved complete anatomic and metabolic responses, consequently precluding any assessment of test performance statistics, such as sensitivity and specificity, of HPV DNA testing for disease surveillance.

Rettig and colleagues (36) showed that whereas plasma cfHPV DNA–guided surveillance facilitates the early detection of recurrences in a subset of patients with HPV-related OPSCC with a median lead-time of 132 days, valid concerns remain about the suboptimal sensitivity of plasma cfHPV DNA testing alone, suggesting a key area for improvement (37). Ultrasensitive next-generation sequencing methods to measure ctDNA, as previously reported in patients with non–small cell lung cancer, is another potentially attractive option that can be leveraged as a dynamic biomarker (38, 39). In addition, a previous study demonstrated a relationship between dynamic changes in peripheral blood neutrophil-to-lymphocyte ratios (NLR) in patients with OPSCC undergoing definitive chemoradiation and statistically significant differences in recurrence-free survival and OS (40). Combining plasma or oral gargle HPV DNA clearance and changes in peripheral blood NLR or other dynamic biomarkers are potential strategies to improve upon plasma or oral gargle HPV DNA alone. For example, one may consider future prospective trials evaluating the potential predictive and prognostic values of a combination of week 4 HPV DNA clearance and reduction in peripheral blood NLR in patients undergoing definitive radiation for HPV-related OPSCC.

Based on our observations of a patient subset demonstrating a transient spike in their week 4 plasma cfHPV DNA levels and another subset with undetectable oral gargle HPV DNA at baseline that later became detectable after treatment initiation, both of which are suggestive of increased HPV DNA shedding due to treatment, we assessed the overlap between these two subsets of patients. However, the overlap between the two groups was low, arguing against a direct relationship between the likelihood of increased treatment-related HPV DNA shedding in plasma and saliva. Furthermore, although limited by the small number of events and follow-up duration, we found no significant association between the transient week 4 plasma cfHPV DNA spike or oral gargle HPV DNA appearance after treatment initiation with end-of-treatment response and 2-year PFS. Therefore, these results suggest that the early transient spike of plasma cfHPV DNA as well as the transient detectability of oral gargle HPV DNA following treatment initiation is of limited prognostic significance.

Our study had several limitations. First, as described in several prior studies, plasma cfHPV DNA testing using ddPCR has an overall sensitivity of 75% to 80% for detecting recurrence (29, 30). Whereas Rettig and colleagues (36) showed that the sensitivity is 91% for patients with known positive baseline cfHPV DNA, recurrences are missed in a substantial proportion of patients. Furthermore, more ultrasensitive methods of minimal residual disease testing via next-generation sequencing such as HPV DeekSeek are being developed, which may improve the performance of minimal residual disease testing (medRxiv: 2025.01.27.25321202; ref. 41). Second, as we used two separate assays for the plasma and oral gargle sample analyses, interpretation of the results specifically regarding the comparison of the kinetics of HPV DNA in the two different bodily fluids should consider this caveat. Third, considering that the patients enrolled in our study were patients with low-risk HPV-related OPSCC who had generally high cure rates, the follow-up for our study was relatively short, and the number of events was very low, limiting our analysis with respect to PFS and OS, including assessment of test performance statistics such as sensitivity and specificity for recurrence detection. In addition, evaluation of the association between cfHPV DNA clearance and end-of-treatment imaging response was not feasible because nearly all patients achieved a complete anatomic and/or metabolic response.

In conclusion, our study demonstrated that week 4 plasma cfHPV DNA clearance was associated with week 4 TTV reduction in early-stage HPV-related OPSCC. A longer follow-up is needed to assess the association between plasma cfHPV DNA clearance and PFS or OS and to determine whether the results will translate to survival endpoints. Future studies aimed at improving cfHPV DNA testing, such as combination dynamic biomarkers or ultrasensitive ctDNA, are warranted.

## Supplementary Material

Supplementary Figure 1 Supplemental Figure 1: Trial Schema and Timepoints of Sample Collection. Oral gargle samples were collected at weekly intervals from baseline to week 4, then at end of treatment and at 3, 6, and 12 months after treatment. Plasma samples were collected at baseline, week 4, end of treatment, and at 3, 6, 12, 18, and 24 months after treatment. Imaging studies done at week 4 was used to assess %reduction of target tumor volume at week 4.

Supplementary Figure 2 Supplemental Figure 2: Association of Baseline Clinical Characteristics with Plasma and Oral Gargle HPV DNA Status. Bar charts show the comparison of proportions of patients with A) detectable versus undetectable baseline plasma cfHPV DNA or B) detectable versus undetectable baseline oral gargle HPV DNA stratified by key baseline clinical variable groups. P-values represent Fisher’s Exact Tests where p < 0.05 is considered significant.

Supplementary Figure 3 Supplemental Figure 3: Week 4 Plasma Cell-Free HPV (cfHPV) or Oral Gargle HPV DNA and its Association with Progression-free Survival (PFS). A) Comparison of PFS for patients with versus without week 4 plasma cfHPV DNA clearance. B) Comparison of PFS for patients with versus without week 4 oral gargle cfHPV DNA clearance. Log-rank test represents a comparison between the respective groups.

Supplementary Figure 4 Supplemental Figure 4: Oral Gargle HPV DNA Collected at Weekly Intervals from Weeks 1 to 4 and Their Associations with Reduction of Target Tumor Volume (TTV) at Week 4. A) Comparison of the reduction of TTV at week 4 in patients with versus without week 4 oral gargle cfHPV DNA clearance; B) week 1 oral gargle cfHPV DNA clearance; C) week 2 oral gargle cfHPV DNA clearance; and D) week 3 oral gargle cefHPV DNA clearance. Positive (i.e. >0) numbers represent tumor volume reduction while negative (i.e. <0) numbers represent tumor volume increase. Statistical test represents Mann-Whitney Test between the 2 groups overlaid by the bolded line. The median and upper and lower quartiles are represented in the bar graphs.

Supplementary Table S1 Supplementary Table S1
