# Supplementary material for: Plasma Cell-Free Human Papillomavirus DNA and Oral Gargle HPV DNA in Patients with HPV-Related Oropharyngeal Cancer Treated with Radiotherapy
Source: Cancer Res Commun. 2025 Jul 22;5(7):1194–202. doi: 10.1158/2767-9764.CRC-25-0180 (PMC12281097; doi:10.1158/2767-9764.CRC-25-0180)
Supplement: Supplementary Figure 1 — Supplemental Figure 1: Trial Schema and Timepoints of Sample Collection. Oral gargle samples were collected at weekly intervals from baseline to week 4, then at end of treatment and at 3, 6, and 12 months after treatment. Plasma samples were collected at baseline, week 4, end of treatment, and at 3, 6, 12, 18, and 24 months after treatment. Imaging studies done at week 4 was used to assess %reduction of target tumor volume at week 4. [file crc-25-0180_supplementary_figure_1_suppsf1.pptx]

## Slide 1
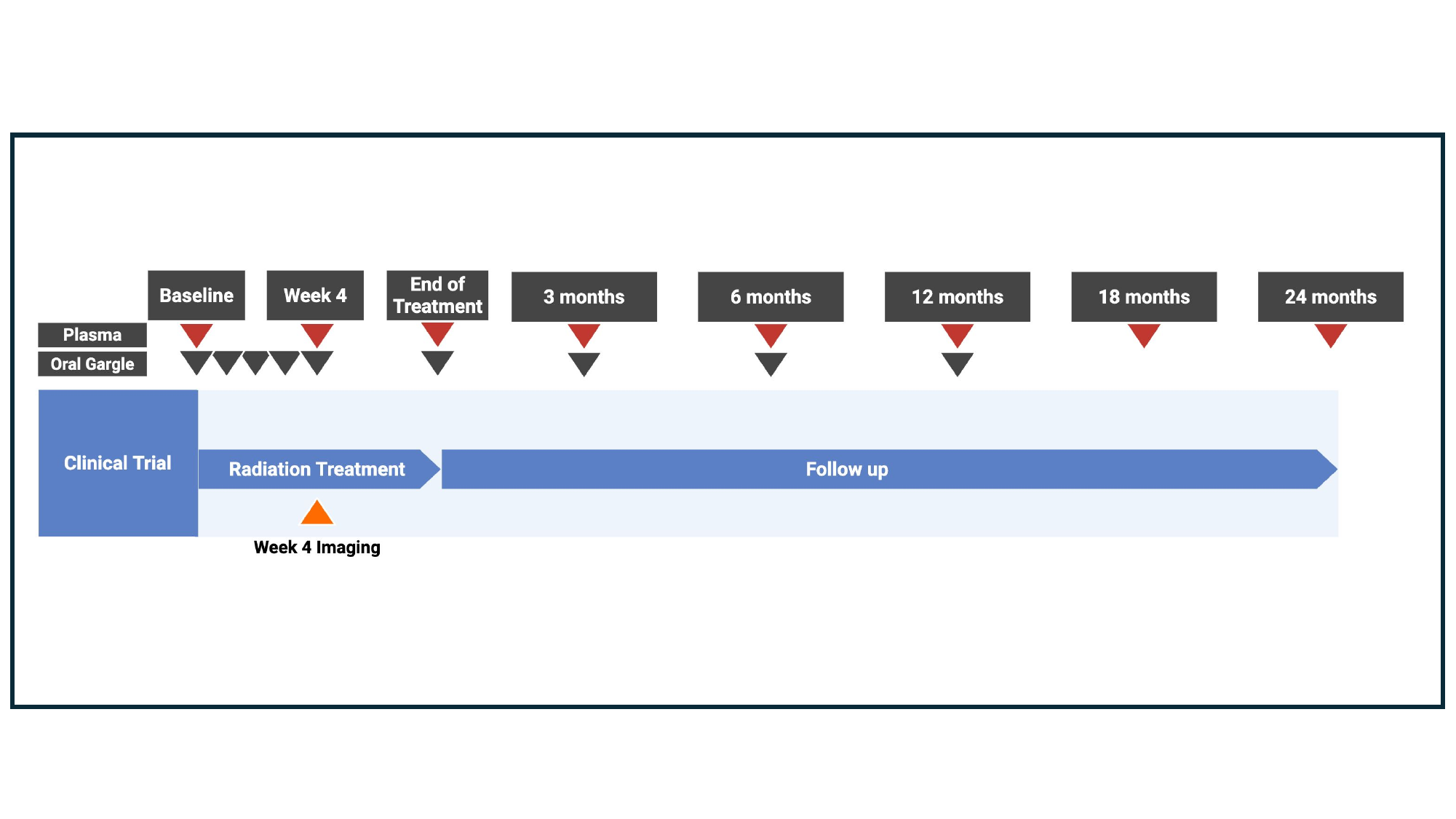

## Slide 2
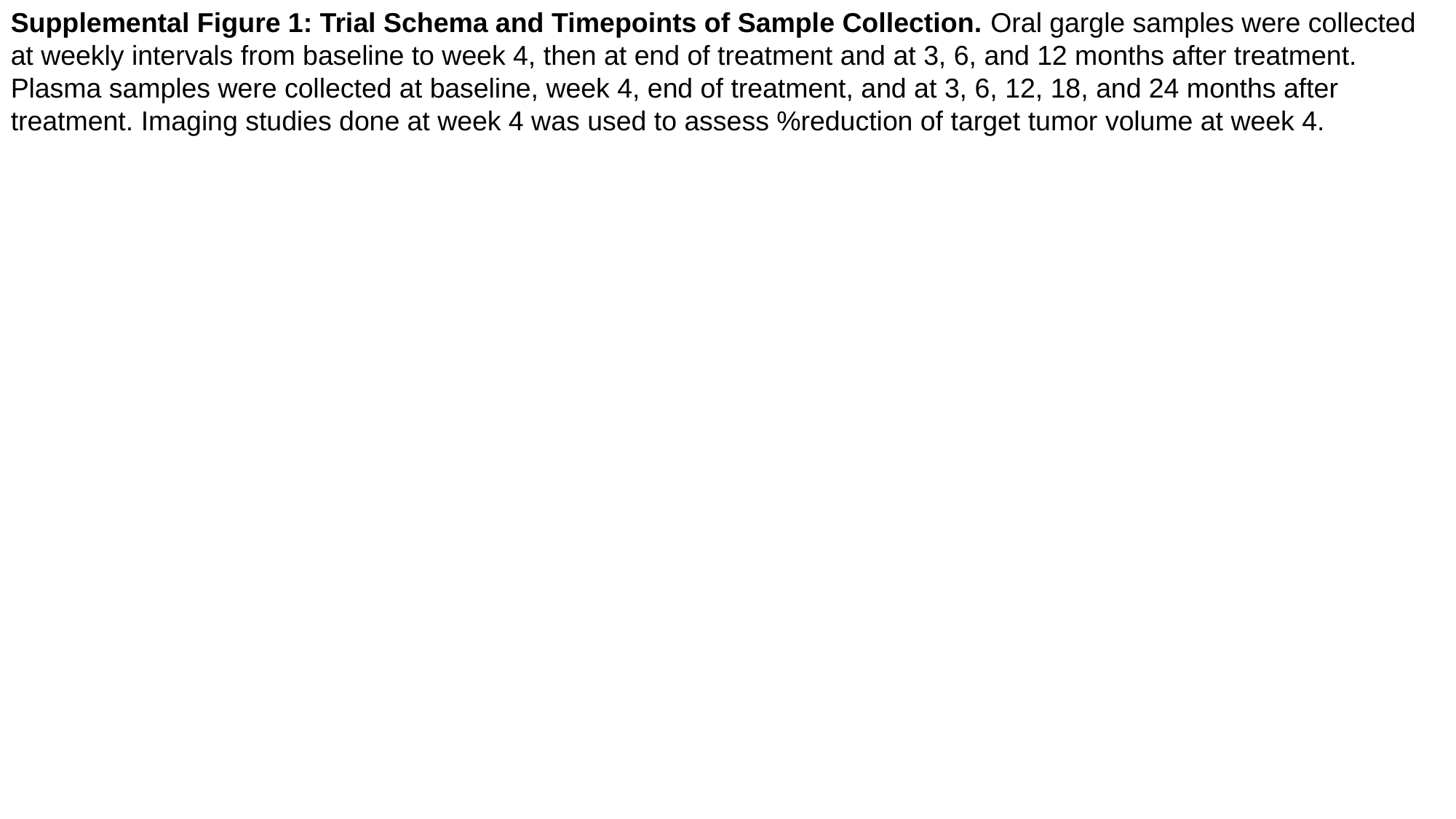

Supplemental Figure 1: Trial Schema and Timepoints of Sample Collection. Oral gargle samples were collected at weekly intervals from baseline to week 4, then at end of treatment and at 3, 6, and 12 months after treatment. Plasma samples were collected at baseline, week 4, end of treatment, and at 3, 6, 12, 18, and 24 months after treatment. Imaging studies done at week 4 was used to assess %reduction of target tumor volume at week 4.
