# Supplementary material for: Plasma Cell-Free Human Papillomavirus DNA and Oral Gargle HPV DNA in Patients with HPV-Related Oropharyngeal Cancer Treated with Radiotherapy
Source: Cancer Res Commun. 2025 Jul 22;5(7):1194–202. doi: 10.1158/2767-9764.CRC-25-0180 (PMC12281097; doi:10.1158/2767-9764.CRC-25-0180)
Supplement: Supplementary Figure 2 — Supplemental Figure 2: Association of Baseline Clinical Characteristics with Plasma and Oral Gargle HPV DNA Status. Bar charts show the comparison of proportions of patients with A) detectable versus undetectable baseline plasma cfHPV DNA or B) detectable versus undetectable baseline oral gargle HPV DNA stratified by key baseline clinical variable groups. P-values represent Fisher’s Exact Tests where p < 0.05 is considered significant. [file crc-25-0180_supplementary_figure_2_suppsf2.pptx]

## Slide 1
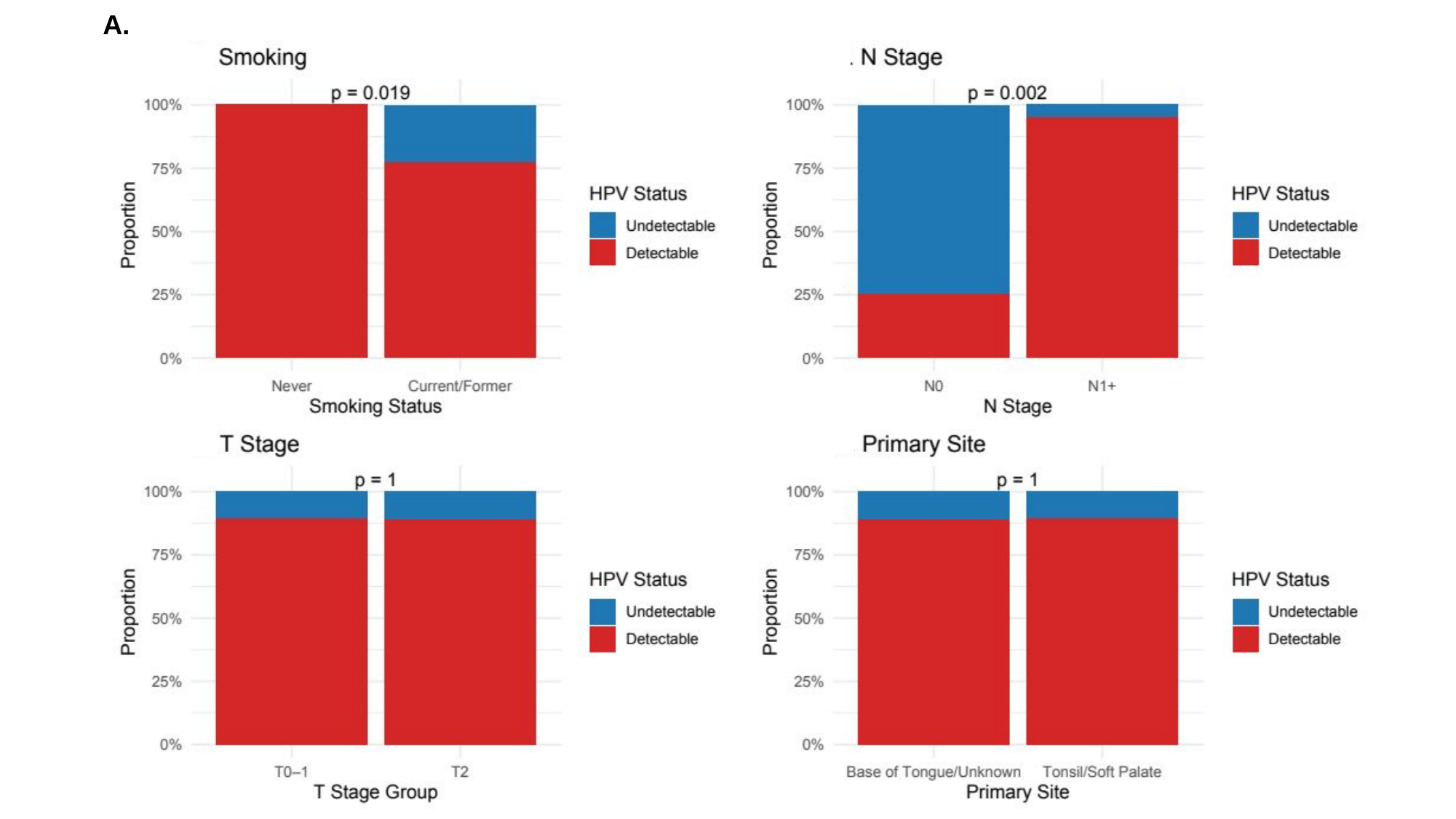

A.

## Slide 2
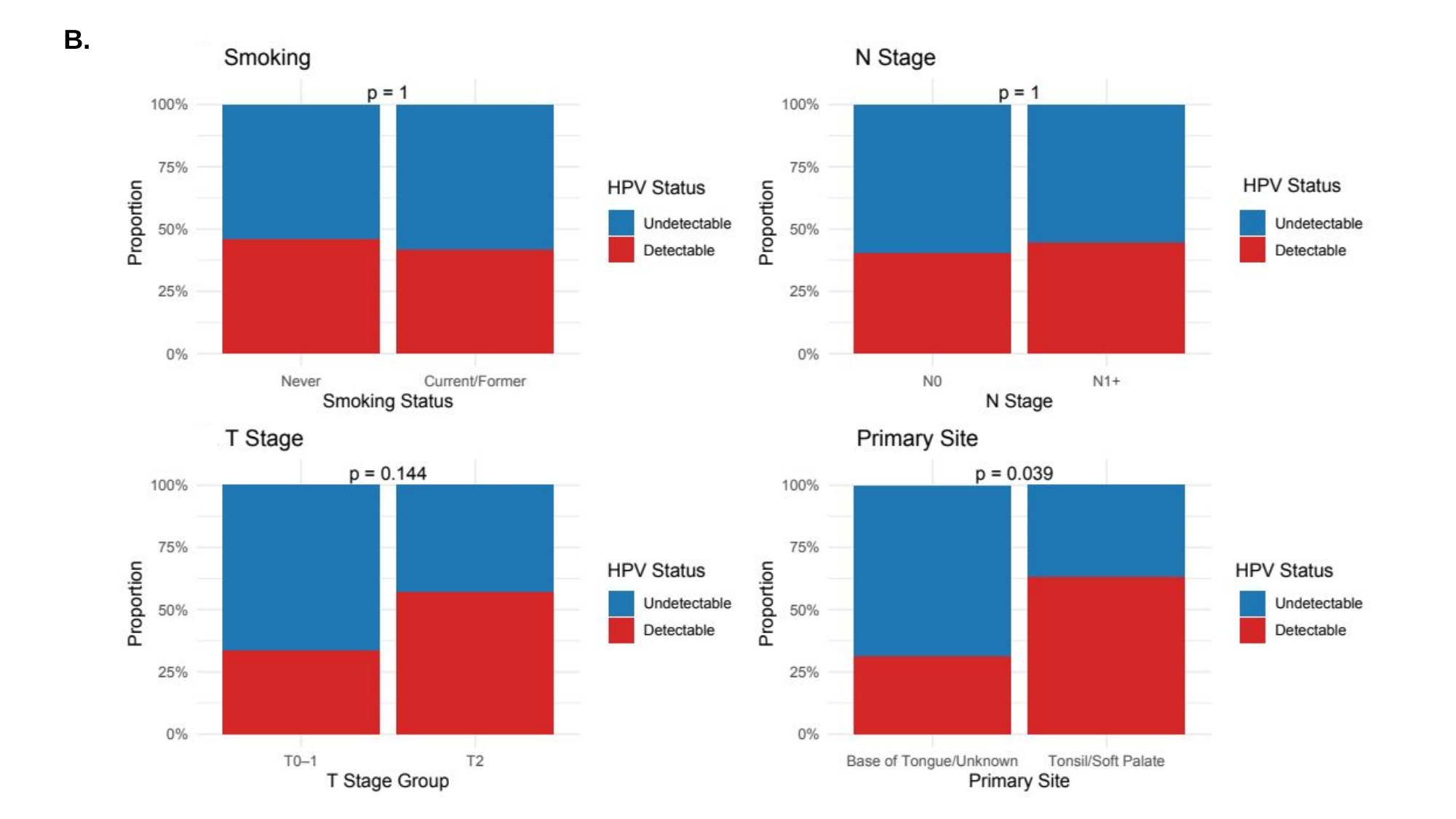

B.

## Slide 3
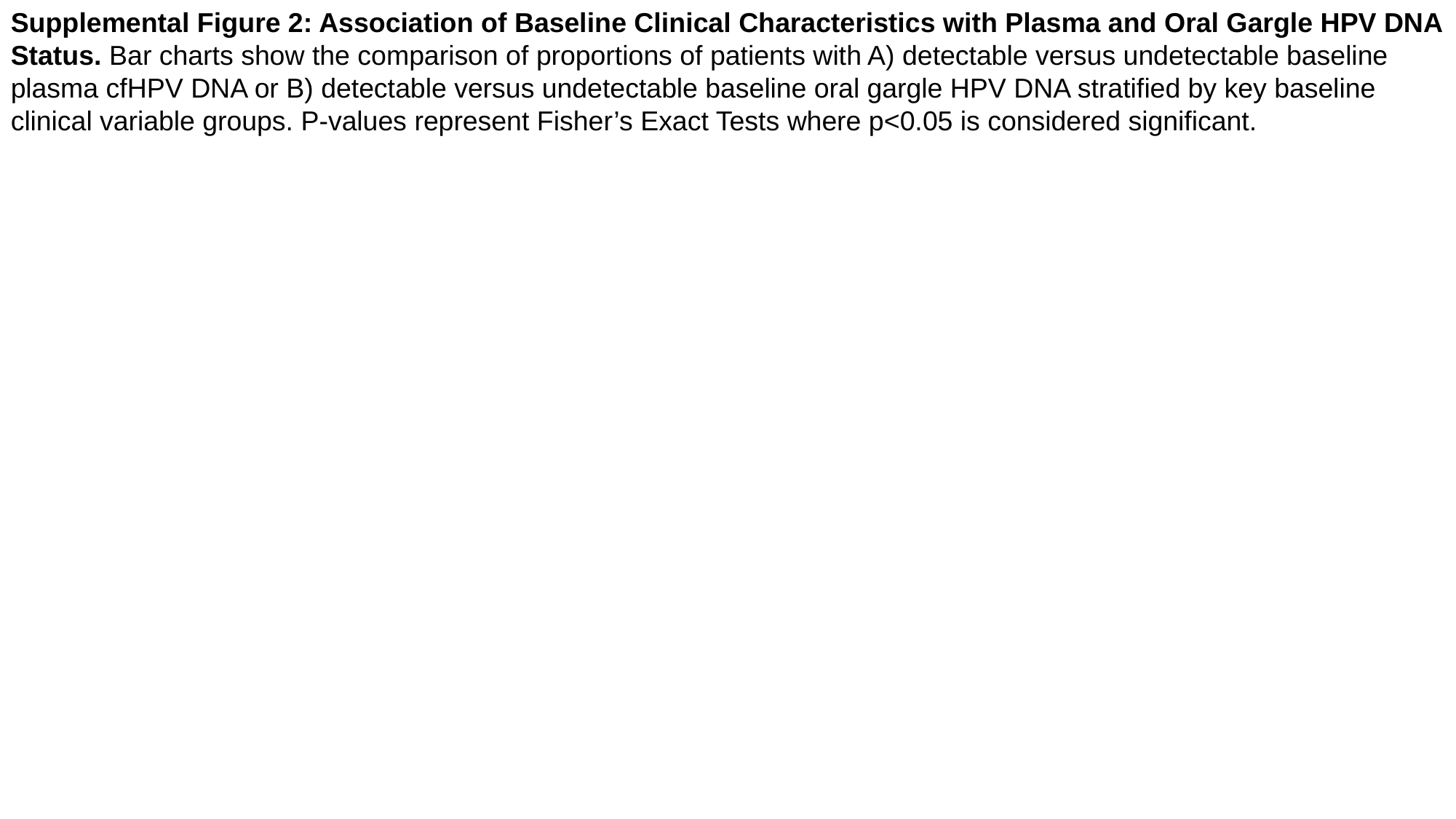

Supplemental Figure 2: Association of Baseline Clinical Characteristics with Plasma and Oral Gargle HPV DNA Status. Bar charts show the comparison of proportions of patients with A) detectable versus undetectable baseline plasma cfHPV DNA or B) detectable versus undetectable baseline oral gargle HPV DNA stratified by key baseline clinical variable groups. P-values represent Fisher’s Exact Tests where p<0.05 is considered significant.
