# Supplementary material for: Plasma Cell-Free Human Papillomavirus DNA and Oral Gargle HPV DNA in Patients with HPV-Related Oropharyngeal Cancer Treated with Radiotherapy
Source: Cancer Res Commun. 2025 Jul 22;5(7):1194–202. doi: 10.1158/2767-9764.CRC-25-0180 (PMC12281097; doi:10.1158/2767-9764.CRC-25-0180)
Supplement: Supplementary Figure 3 — Supplemental Figure 3: Week 4 Plasma Cell-Free HPV (cfHPV) or Oral Gargle HPV DNA and its Association with Progression-free Survival (PFS). A) Comparison of PFS for patients with versus without week 4 plasma cfHPV DNA clearance. B) Comparison of PFS for patients with versus without week 4 oral gargle cfHPV DNA clearance. Log-rank test represents a comparison between the respective groups. [file crc-25-0180_supplementary_figure_3_suppsf3.pptx]

## Slide 1
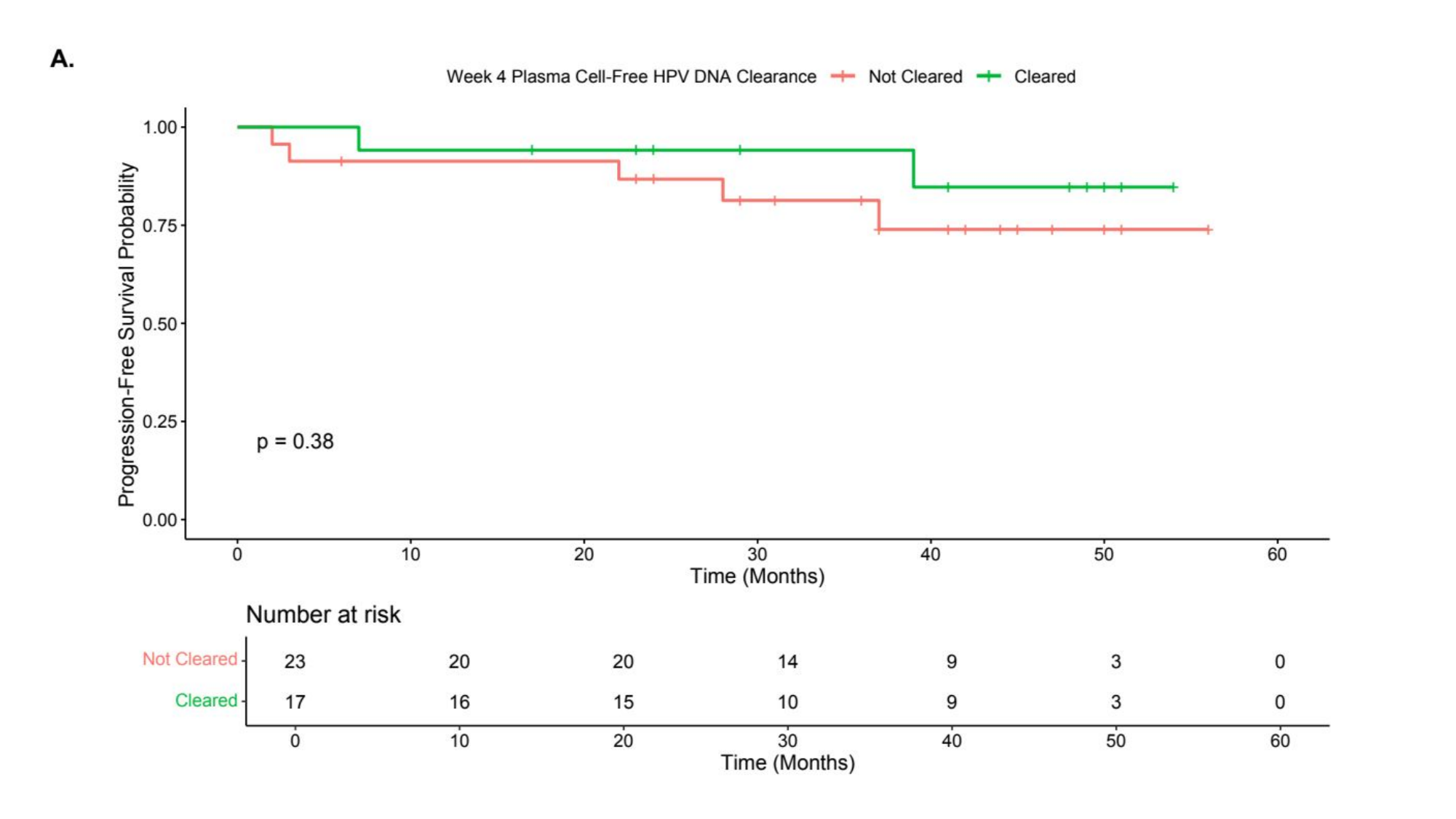

## Slide 2
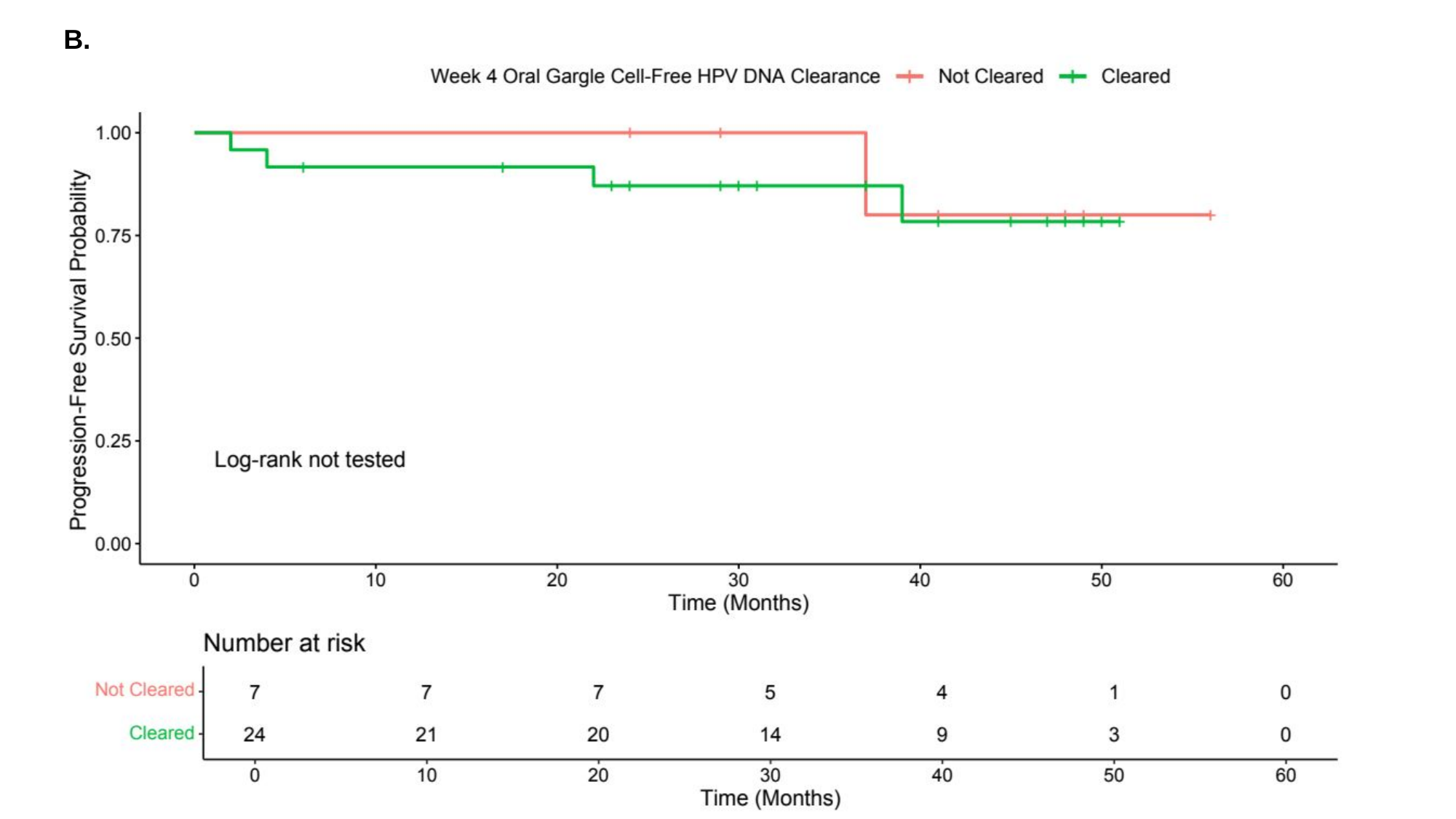

B.

## Slide 3
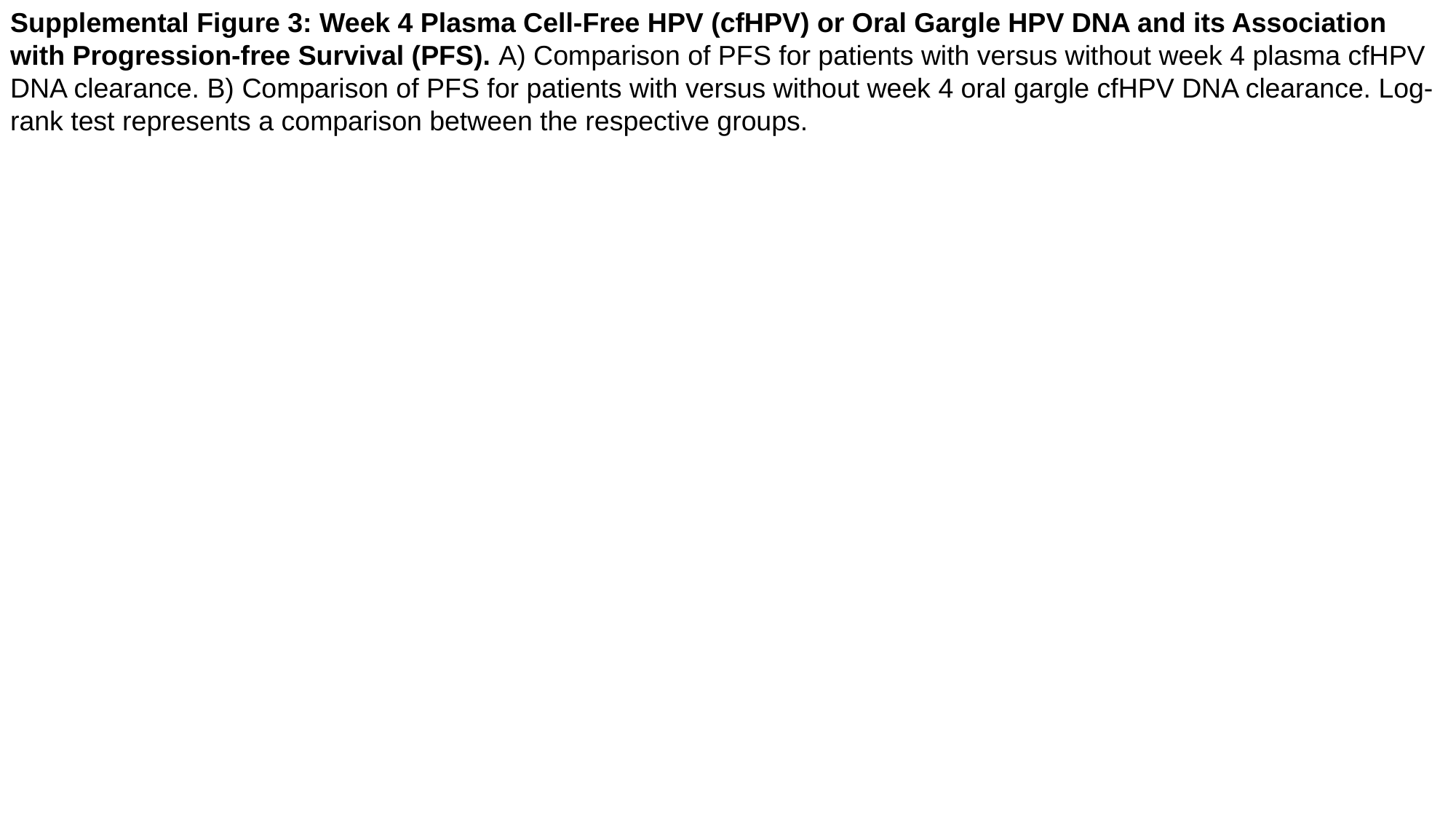

Supplemental Figure 3: Week 4 Plasma Cell-Free HPV (cfHPV) or Oral Gargle HPV DNA and its Association with Progression-free Survival (PFS). A) Comparison of PFS for patients with versus without week 4 plasma cfHPV DNA clearance. B) Comparison of PFS for patients with versus without week 4 oral gargle cfHPV DNA clearance. Log-rank test represents a comparison between the respective groups.
