# Supplementary material for: Plasma Cell-Free Human Papillomavirus DNA and Oral Gargle HPV DNA in Patients with HPV-Related Oropharyngeal Cancer Treated with Radiotherapy
Source: Cancer Res Commun. 2025 Jul 22;5(7):1194–202. doi: 10.1158/2767-9764.CRC-25-0180 (PMC12281097; doi:10.1158/2767-9764.CRC-25-0180)
Supplement: Supplementary Figure 4 — Supplemental Figure 4: Oral Gargle HPV DNA Collected at Weekly Intervals from Weeks 1 to 4 and Their Associations with Reduction of Target Tumor Volume (TTV) at Week 4. A) Comparison of the reduction of TTV at week 4 in patients with versus without week 4 oral gargle cfHPV DNA clearance; B) week 1 oral gargle cfHPV DNA clearance; C) week 2 oral gargle cfHPV DNA clearance; and D) week 3 oral gargle cefHPV DNA clearance. Positive (i.e. >0) numbers represent tumor volume reduction while negative (i.e. <0) numbers represent tumor volume increase. Statistical test represents Mann-Whitney Test between the 2 groups overlaid by the bolded line. The median and upper and lower quartiles are represented in the bar graphs. [file crc-25-0180_supplementary_figure_4_suppsf4.pptx]

## Slide 1
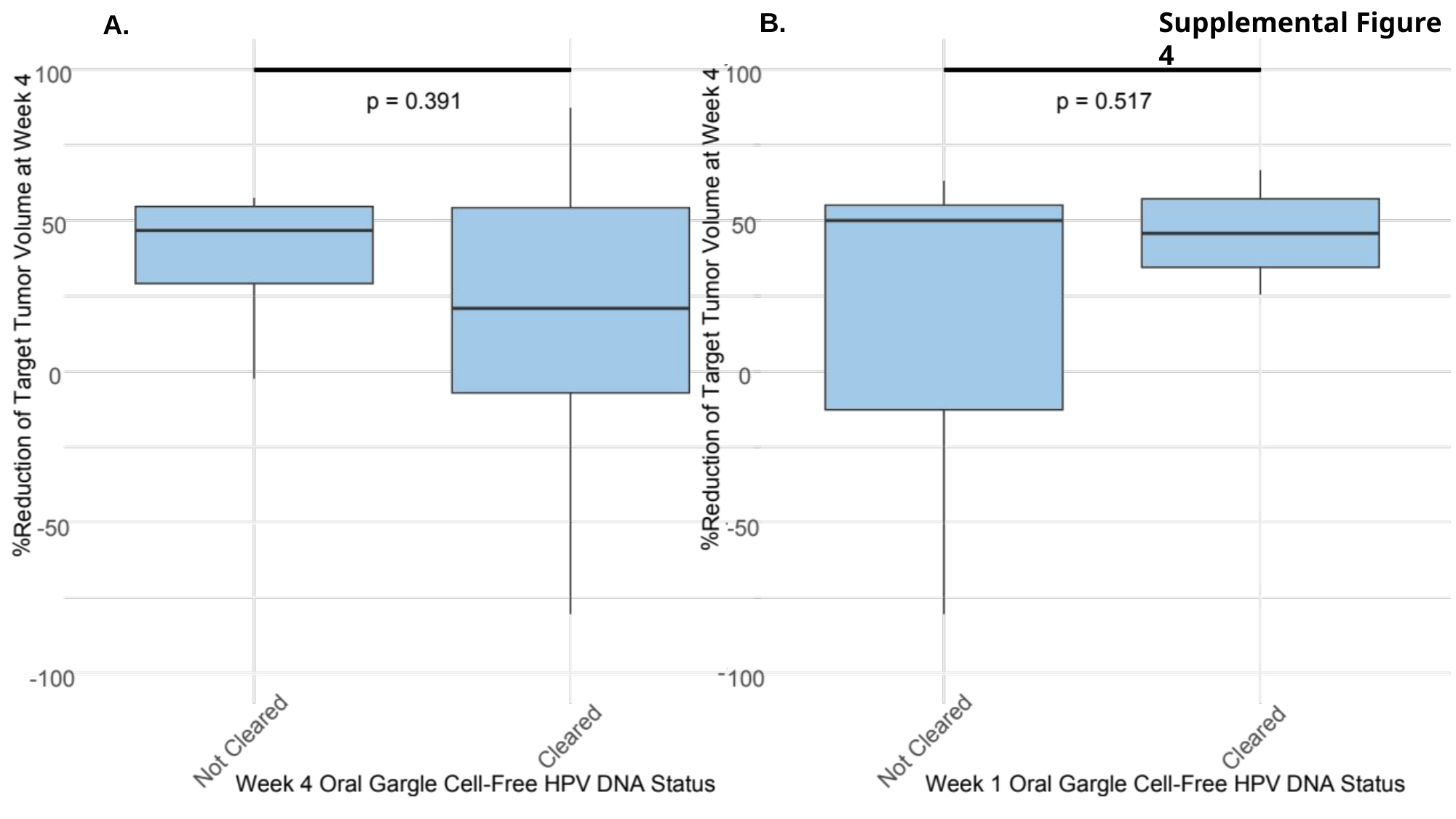

B.
Supplemental Figure 4
A.

## Slide 2
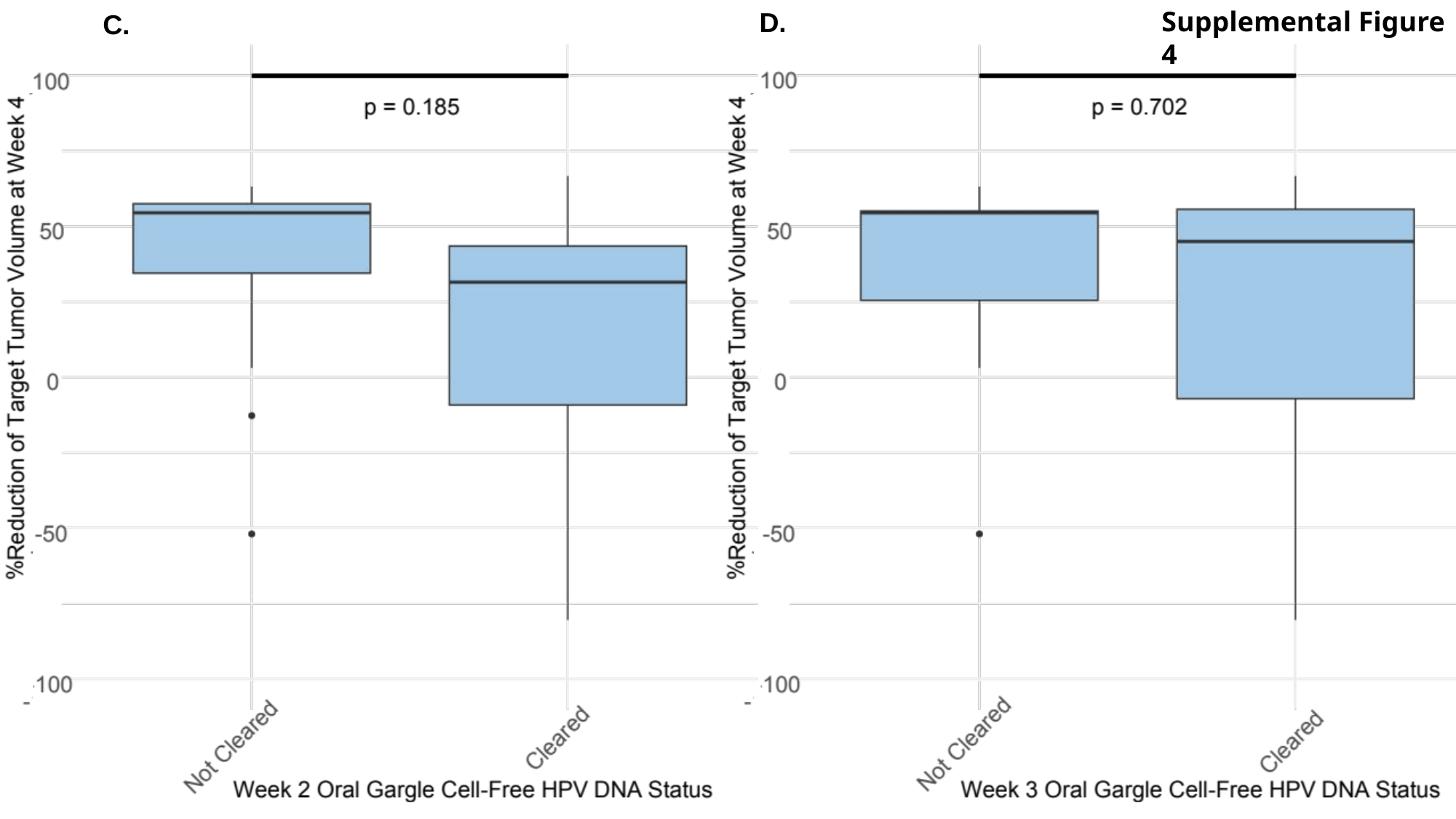

D.
Supplemental Figure 4
C.

## Slide 3
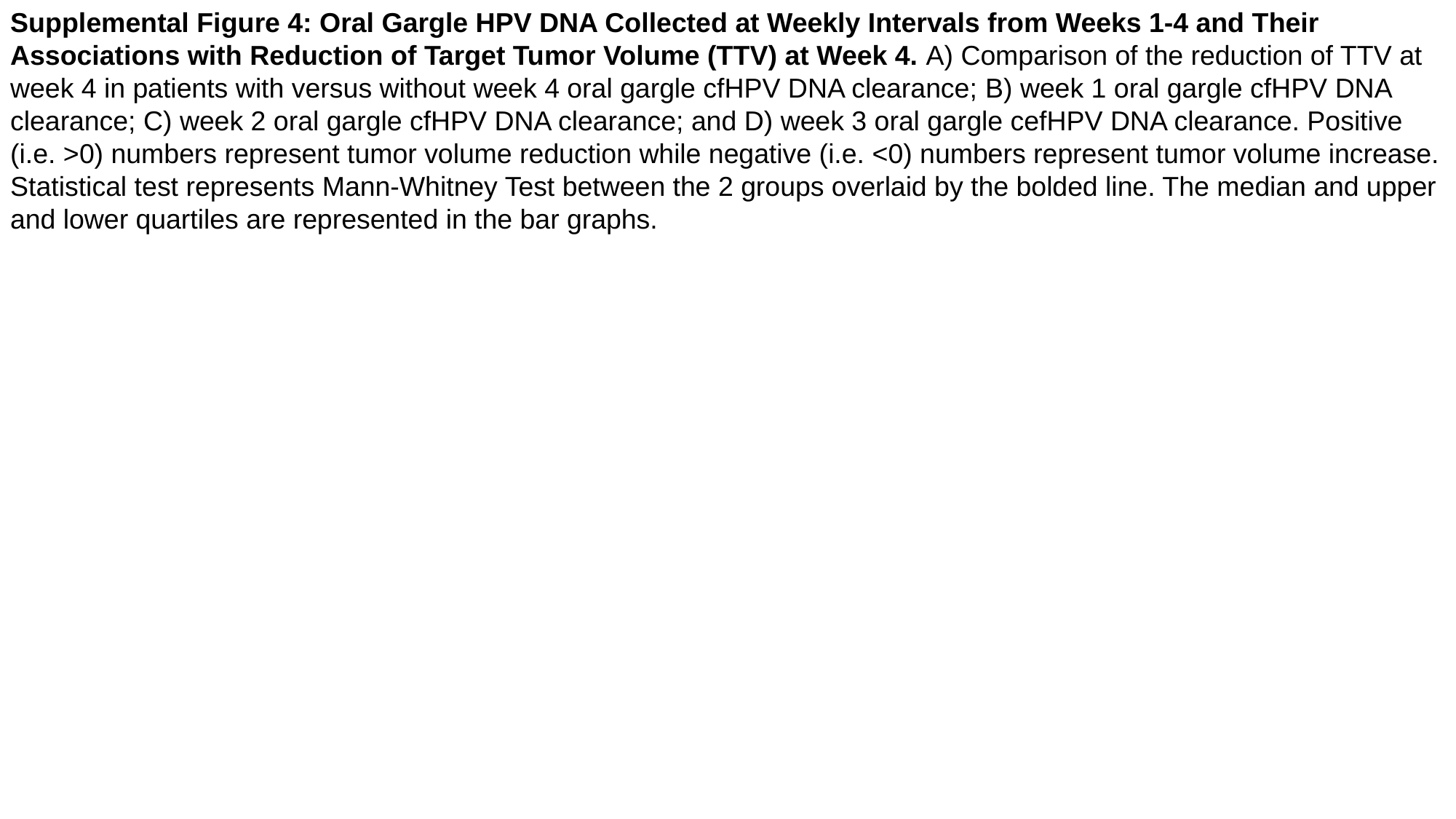

Supplemental Figure 4: Oral Gargle HPV DNA Collected at Weekly Intervals from Weeks 1-4 and Their Associations with Reduction of Target Tumor Volume (TTV) at Week 4. A) Comparison of the reduction of TTV at week 4 in patients with versus without week 4 oral gargle cfHPV DNA clearance; B) week 1 oral gargle cfHPV DNA clearance; C) week 2 oral gargle cfHPV DNA clearance; and D) week 3 oral gargle cefHPV DNA clearance. Positive (i.e. >0) numbers represent tumor volume reduction while negative (i.e. <0) numbers represent tumor volume increase. Statistical test represents Mann-Whitney Test between the 2 groups overlaid by the bolded line. The median and upper and lower quartiles are represented in the bar graphs.
