# Supplementary material for: Plasma Cell-Free Human Papillomavirus DNA and Oral Gargle HPV DNA in Patients with HPV-Related Oropharyngeal Cancer Treated with Radiotherapy
Source: Cancer Res Commun. 2025 Jul 22;5(7):1194–202. doi: 10.1158/2767-9764.CRC-25-0180 (PMC12281097; doi:10.1158/2767-9764.CRC-25-0180)
Supplement: Supplementary Table S1 [file crc-25-0180_supplementary_table_s1_suppst1.docx]

| **Supplementary Table S1.** Representativeness of Study Participants | |
| --- | --- |
| Cancer type(s)/subtype(s)/stage(s)/condition | Head and neck squamous cell carcinoma (HNSCC), in particular, HPV-related oropharyngeal squamous cell carcinoma (OPSCC) |
| Considerations related to: | |
| Sex | Males are affected significantly more than females with a 4:1 ratio. The incidence rate of HNSCC as well as HPV-related OPSCC in males is approximately 18 per 100,000 and 8 per 100,000 in the US, respectively. |
| Age | The median age of individuals diagnosed with HNSCC as well as HPV-related OPSCC is 66 and 55-60, respectively. |
| Race/ethnicity | In the US, the incidence of HPV-related OPSCC is highest in Non-Hispanic White individuals. While incidence is lower in Non-Hispanic Black individuals, outcomes are the worst. Asian and Pacific Islanders have the lowest incidence. |
| Geography | In the United States, head and neck cancer accounts for 3% of malignancies, with approximately 66,000 cases annually and 15,000 deaths. Worldwide, the highest incidence of HPV-related OPSCC is in the North American and Western European countries, while Eastern European and Asian countries have lower incidences. |
| Other considerations | Our study was a single-center study in an academic institution in the Southeastern Region of the US, where incidence of HPV-related OPSCC among the highest. |
| Overall representativeness of this  study | The median age of our study population (66) as well as the male-to-female ratio (4:1) is similar to that of the median age of the general HPV-related OPSCC patient population in the US. |
